# Real time imaging of single extracellular vesicle pH regulation in a microfluidic cross-flow filtration platform

**DOI:** 10.1038/s42003-021-02965-7

**Published:** 2022-01-10

**Authors:** Vladimir Riazanski, Gerardo Mauleon, Kilean Lucas, Samuel Walker, Adriana M. Zimnicka, James L. McGrath, Deborah J. Nelson

**Affiliations:** 1 Department of Pharmacological and Physiological Sciences, The University of Chicago, Chicago, IL 60637 USA; 2 Department of Biomedical Engineering, University of Rochester, Rochester, NY 14627 USA

**Keywords:** Super-resolution microscopy, Nanoparticles

## Abstract

Extracellular vesicles (EVs) are cell-derived membranous structures carrying transmembrane proteins and luminal cargo. Their complex cargo requires pH stability in EVs while traversing diverse body fluids. We used a filtration-based platform to capture and stabilize EVs based on their size and studied their pH regulation at the single EV level. Dead-end filtration facilitated EV capture in the pores of an ultrathin (100 nm thick) and nanoporous silicon nitride (NPN) membrane within a custom microfluidic device. Immobilized EVs were rapidly exposed to test solution changes driven across the backside of the membrane using tangential flow without exposing the EVs to fluid shear forces. The epithelial sodium-hydrogen exchanger, NHE1, is a ubiquitous plasma membrane protein tasked with the maintenance of cytoplasmic pH at neutrality. We show that NHE1 identified on the membrane of EVs is functional in the maintenance of pH neutrality within single vesicles. This is the first mechanistic description of EV function on the single vesicle level.

## Abstract (web summary)

Riazanski et al describe a platform to capture extracellular vesicles (EVs) using a nanoporous silicon nitride membrane, investigate the expression of NHE1 protein on the surface of EVs and monitor the transport of Na+ and H+ at the single EV level. The authors report a mechanistic function of the proteins found in EVs and specifically identify NHE1 on a single EV, where it maintains pH neutrality within single vesicles.

## Introduction

Extracellular vesicles (EVs) are likely to mirror their intracellular cousins in the expression of surface transport proteins that define their characteristic function and perhaps cellular origin. For example, the packaging of neurotransmitters into synaptic vesicles is accomplished by the vesicular GABA and glutamate transporters using the electrochemical proton gradient of the vesicular V-ATPase^1^. Hormones are processed in secretory granules in pancreatic islet cells also via an acidification step requiring both anion channels and V-ATPase activity. It is then reasonable to assume that the presence of surface transporters and channels on EVs also contributes to a crucial step in the maintenance or processing of luminal cargo. Perhaps the most important function of EVs, is envisioned as a nanoparticle platform for therapeutically relevant gene and drug delivery approaches with attention devoted to how they might be engineered for targeted delivery and their cargo protected from environmentally induced modification^2–7^.

A large literature is devoted to the characterization of EV-associated microRNAs (miRNAs), small non-coding RNA molecules that play an important role in disease pathogenesis with a focus on cancer and how these gene regulatory elements might be exported in EVs from specific populations of cells^8^. EVs and their cargo have been identified diversely as biomarkers for disease as well as effective vehicles for molecular target therapies. Independent of their identified function, either as genetic signaling elements or genetic delivery vehicles, maintenance of a stable pH in the lumen of vesicles, both microvesicles derived from a plasma membrane scission and exosomes secreted from endocytically derived multivesicular bodies (MVBs), is essential.

The burgeoning literature characterizing the functional role as well as the derivation of EVs is bereft of tools that would allow the investigator to examine vesicles individually and determine the molecular identity of transport proteins on isolated vesicles and whether they are functional. To date, vesicles have been isolated and identified by size using a variety of biochemical techniques. Transmission electron microscopy as well cryo-techniques demonstrate that isolated EVs are highly heterogeneous in size but with no direct connection between size and content. Thus, the current studies provide a new platform to isolate stabilized single vesicles and study possible mechanisms for cargo loading and maintenance.

Mechanistically, vesicle-associated transporter function is generally studied in preparations that are amenable to bulk analysis, e.g. neuronal synaptic vesicles^9–11^, chromaffin cell catecholamine containing granules^12^, or hormone-containing secretory granules^13^. This is not the case with EVs. EVs isolated from either cultured cells or primary bodily fluid samples yield heterogeneous preparations with potentially important subclasses that may go undetected in a bulk analysis of transport. The focus of our investigation is on small EVs which are certainly heterogeneous but known to be of biologic and therapeutic importance. The advantage of our investigation is an assessment of the functional properties of protein at single EV levels. Using a microfluidic platform, we developed an approach to allow single EV visualization and ion-sensitive dye measurement. After selectively capturing secreted EVs in the pores of ultrathin nanoporous silicon nitride (NPN) membranes based on their size, we use real-time fluorescence imaging to measure the kinetics of pH changes in single vesicles brought about by the activity of the Na/H antiporter.

## Results

### Construction of the membrane-based platform used to stabilize and visualize isolated EVs

The development of a microfluidics platform for the stabilization and sequential superfusion of single EVs allowed us to perform mechanistic studies on vesicle transport proteins. EVs have not been characterized as to possible transport proteins that might serve to stabilize vesicle payload in an array of fluid environments. In fact, the determination of the vesicle lumen pH, not determined to date, was the impetus for the current investigation.

**Fig. 1:**
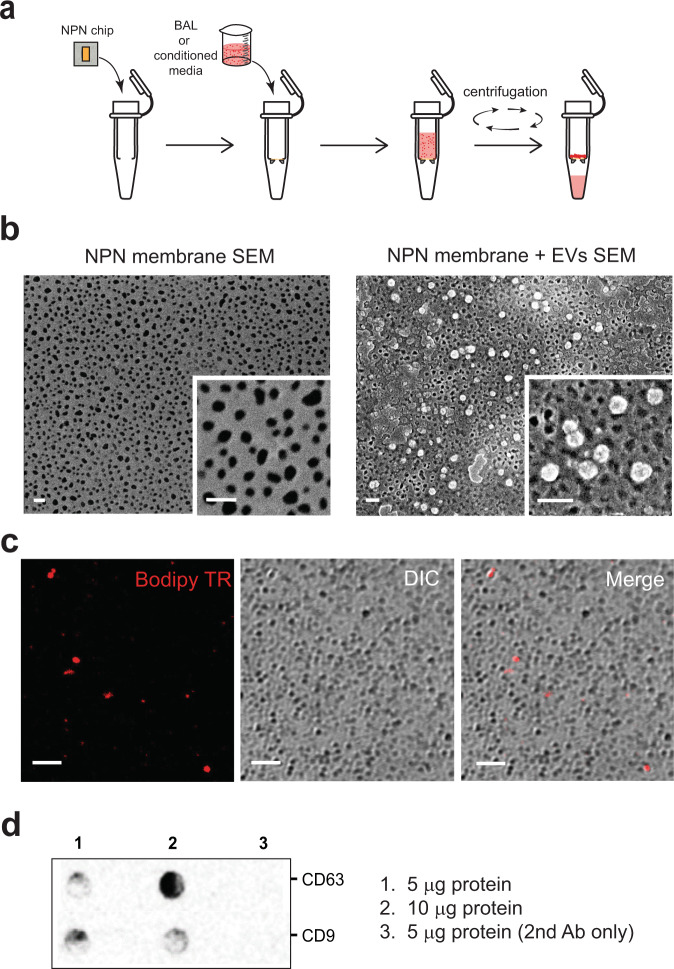
Capture and characterization of EVs on NPN membranes. **a** Steps involved in the capture of EVs from the pre-cleared BAL or cell-conditioned media, using SepCon™ centrifuge tubes with a plasma-cleaned NPN chip insert. The insert consists of an ultrathin nanoporous NPN membrane and silicone support. **b** SEM images showing the nanoporous membrane, before (left) and after (right) filtration of EV-rich fluids (scale bar 200 nm). **c** Laser confocal microscopic images of NPN membrane-captured particles stained with the lipid dye, Bodipy-Texas Red (Bodipy TR) in Differential Interference Contrast (DIC) mode and merged image of two channels (Merge). Scale bar 5 µm. **d** A dot blot analysis of NPN-captured vesicles verifies the expression of CD9 and CD63 tetraspanin proteins.

Vesicles in pre-cleared isolation solution from either cell culture media or murine bronchoalveolar lavage (BAL) following high-speed centrifugation steps were loaded onto the NPN membranes by dead-end filtration using SepCon® spin columns specially designed to accommodate the “membrane chip” (Fig. 1a). Free-standing NPN membranes are patterned into the central portions of the silicon chip which enables ready extraction from the SepCon® following vesicle capture and safe handling of the 100 nm thick membranes during transfer to the microfluidic system. The steps of NPN membrane chip loading with vesicles are illustrated in Fig. 1a. Initially, the capability of NPN membranes to trap and retain vesicles that do not pass through its nanopores with a mean diameter 50 nm^14^ was tested by the application of commercially available plasma-derived EVs (Hansa Biomed), and visualization with scanning electron microscopy (SEM) (Fig. 1b). For functional characterization of EVs, lung-derived vesicles were visualized in confocal microscopy with the lipid fluorescent label Bodipy TR (1 µM) as seen in Fig. 1c for a BAL sample. Although we are unable to determine how many of the apparent vesicles (seen as Bodipy TR spots) we capture on the NPN are actually small EVs, evidence that the BAL sample contained EVs was obtained in dot blots probed with the tetraspanins CD63 and CD9 commonly associated with EV populations (Fig. 1d).

**Fig. 2:**
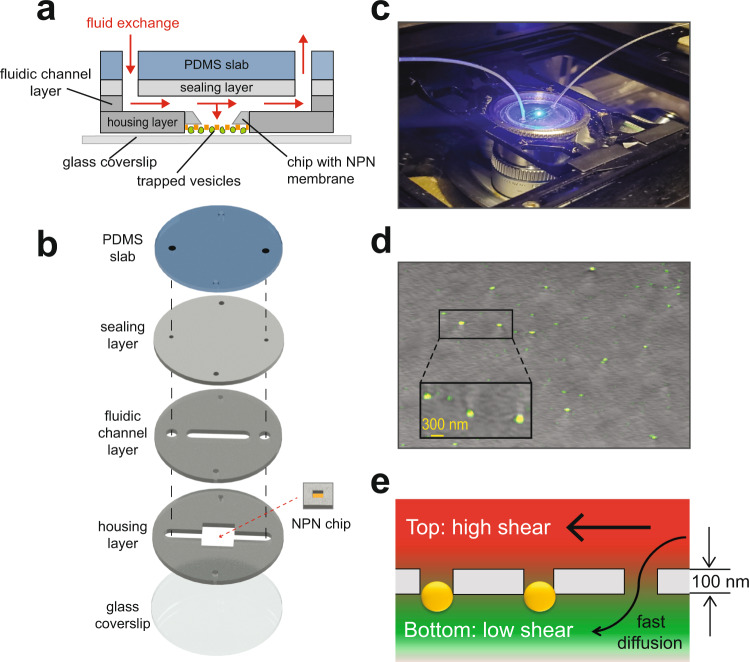
NPN membrane-based platform for investigation of EV properties in real time. **a** Schematic cross-sectional diagram of the microfluidic device used to study functional responses of individual, membrane-stabilized EVs in response to changes in ionic composition of external solution. **b** Stack of PDMS layers comprising the device, including the housing layer patterned for insertion of the NPN chip. **c** Photograph of the device assembled on the stage of laser scanning confocal microscope. Inlet and outlet tubing allowed for the flow of solutions over the NPN membrane-immobilized vesicles, at a rate of 5–10 µl/min, maintained by a pressure-driven microfluidic flow system (ElveFlow®). **d** Image of fluorescent beads (100 nm in diameter), trapped and retained on the NPN membrane under conditions of tangential flow (scale bar 300 nm). **e** Schematic depiction of how the ultrathin (100 nm thick) nanoporous NPN membrane (gray) separates perfusion flows: top compartment with high shear force provides fast solution exchange (red) which diffuses rapidly through nanopores to the bottom compartment where trapped vesicles (yellow) are exposed to low shear force (green).

The platform used to house and superfuse the NPN-membrane-captured individual vesicles was drawn from the studies of Dehghani et al.^15^ and is schematized in Fig. 2a. The multi-layered platform is sealed to a glass coverslip base and consists of a polydimethylsiloxane (PDMS) slab, sealing layer, a fluidic channel layer, and a housing layer in which the ultrathin (100 nm thick) NPN membrane is housed (Fig. 2b). The vesicle-loaded side of the NPN chip is placed in the housing layer in contact with a plasma cleaned coverslip. This configuration provides a minimal optical working distance and allows for fluid flow to occur on the backside of the membrane without disturbing captured vesicles. Given the strong fluid decoupling between the top and bottom of the membrane^16^, reagents move into and out of the vesicle population by diffusion through the membrane’s pores. The entire device is placed on the microscope stage (Fig. 2c) which then allows visualization of Bodipy-labeled vesicles trapped within and atop the membrane pores. The retention of trapped nanoparticles during the chip superfusion was confirmed by loading onto the platform and confocal imaging of 100 nm fluorescent beads (Fig. 2d). The distribution of the superfusion fluid flow force on the captured particles in the device when placed on the microscope stage is illustrated in Fig. 2e.

### Preparations used in the isolation and characterization of functional EV protein expression

In order to explore the generality of sodium–hydrogen exchanger (NHE) expression and function in EVs, we examined vesicles obtained and isolated from multiple cultured cell types. In addition, we used murine BAL samples that contained vesicles derived from heterogeneous cell types in the pulmonary tree. EVs were isolated using both size-exclusion chromatography as well as asymmetric flow field-flow fractionation (AF4) (see Figs. S1 and S2) prior to loading onto the NPN membranes. Our experiments were designed to test the platform’s capacity to capture and functionally assess EV function in media from homogeneous cultured cells as well as in a complex biofluid as represented by BAL. The EV census in BAL was considerably greater than that derived from cultured cells. While the EV origin was unknown in the BAL samples, they were likely to be of both epithelial as well as pulmonary immune cell origin, i.e. alveolar macrophages or neutrophils that are plentiful in the pulmonary tree.

### Optical determination of single EV size using super-resolution microscopy: comparison to sized fluorescent beads

**Fig. 3:**
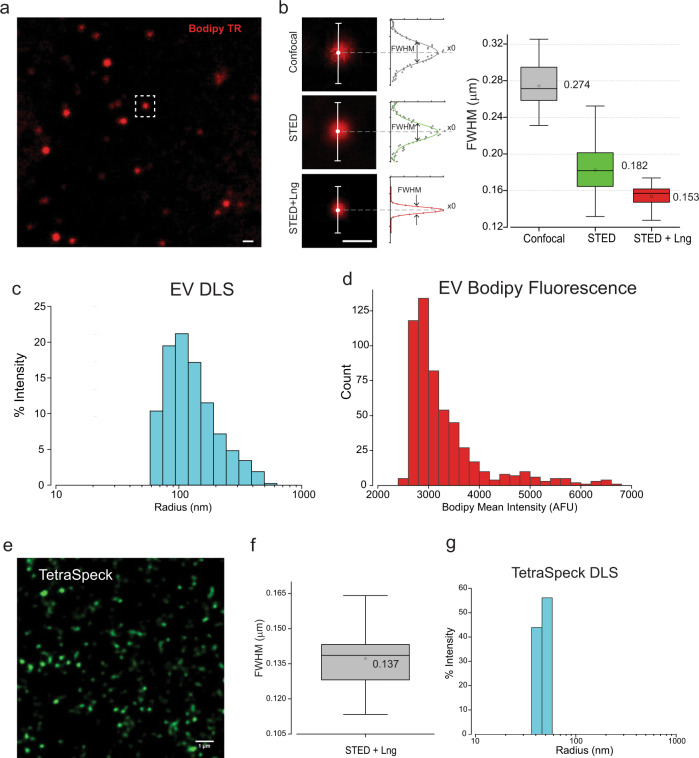
Single vesicle resolution on NPN membrane with different nanoparticle measurement techniques. **a** Image of NPN membrane-captured particles stained with Bodipy-TR (scale bar 0.5 µm, insert box—a spot shown in **b**). **b** Left panel. A representative analysis of a fluorescent spot image analysis using line cross-section intensity analysis with Gaussian fitting (middle panel) demonstrating the relative resolutions of the three modes of imaging: Confocal, STED, and STED + Lng. Normalized to the peak data points shown by dots, with Gaussian fits as the solid lines. Right panel. The Gaussian FWHM analysis statistic box plot obtained for Confocal (*n* = 31, mean 274 ± 4 nm), STED (*n* = 27, mean 182 ± 5 nm), and STED + Lng (*n* = 13, mean 153 ± 4 nm). **c** Distribution plot of AF4 isolated EV hydrodynamic radius of DLS measurements in bulk solution. **d** NPN membrane captured Bodipy TR-labeled EVs fluorescence mean intensities distribution plot (*n* = 554 spots). **e** Image of NPN membrane-captured TetraSpeck^TM^ 0.1 µm fluorescent microspheres obtained with green 505/515 nm excitation/emission (scale bar 1 µm). **f** Gaussian FWHM analysis statistic box plot obtained for TetraSpeck^TM^ microspheres using STED + Lng (*n* = 15, mean 137 ± 3 nm). **g** Distribution plot of TetraSpeck^TM^ microsphere hydrodynamic radius of DLS measurements in bulk solution.

Our experimental goal was the visualization and functional interrogation of single EVs isolated from BAL and commonly used cell lines. This obligated determining how a single vesicle was defined optically when stabilized in our permeable, membrane-based platform. Could we distinguish between a single labeled vesicle and a labeled structure formed by two contiguous but separate vesicles? This is a non-trivial problem given the fact that EVs are heterogeneous in size across a large range of 30–250 nm depending upon source and isolation technique. We began by comparing images of Bodipy TR-labeled vesicles using three different imaging techniques (Fig. 3a, b). Line cross-section plots across Bodipy TR labeled fluorescent spots yielded in intensity profile plots that were fitted using a Gaussian fitting function in order to determine the spot full width at half maximum (FWHM). Analysis of typical confocal microscopic images (Fig. 3b (left panel, confocal) yielded a Gaussian distribution of intensities with a mean FWHM of 274 nm (Fig. 3b, (right panel, gray box). When the identical particles were imaged using stimulated emission depletion (STED) super-resolution imaging (Fig. 3b, STED) microscopy and analyzed, a Gaussian distribution of intensities resulted in a mean FWHM of 182 nm. STED imaging combined with the Leica LIGHTNING image deconvolution techniques (Fig. 3b, STED + Lng) yielded a Gaussian FWHW mean of 153 nm, which corresponds to the highest level of resolution obtainable with our Leica SP8-STED light microscopy (see below). The Gaussian distribution for each of the three techniques remained a single distribution suggesting that the vesicle intensities were not the result of integral multiples of aggregated particles or fusion of multiple vesicles. Measurements of EV size distribution in the bulk sample using dynamic light scattering (DLS) (Fig. 3c) confirmed our observation of a broad EV size range and that the NPN membrane is capable of capturing the narrow EV sizes as well. The sample DLS measurement was indeed a single broad distribution with a large diameter tail, an observation that was identical to the distribution of EV Bodipy fluorescent intensity distribution seen in Fig. 3d (note the DLS measurements are for particle radius). We benchmarked our observations on isolated EVs using TetraSpeck^TM^ 100 nm fluorescent beads for comparison. In Fig. 3e, fluorescent beads were imaged using STED microscopy with deconvolution and the population size was analyzed as in Fig. 3b for the isolated EV particles from BAL. While DLS measurements indicated that the TetraSpeck™ beads were monodisperse and ~94 nm in diameter, the mean size of these particles in optical images was 137 nm. This comparison indicates that the optical resolution of the Leica SP8-STED after deconvolution is effectively the same value as our size measurements for EVs (137 ± 3 versus 153 ± 4 nm). Thus, to within the optical resolution of our imaging system, we conclude that our images of NPN-captured EVs are single vesicles.

### Double staining EVs with indicators for content and pH

After establishing that the NPN membrane can capture single EV vesicles in a specific size range (around diameter 100 nm), we continued with functional characterization of the vesicular transport system. Acridine orange (AO) was chosen as a pH indicator since it has been used commonly for determining pH changes in a wide range of submicron size intracellular organelles including lysosomes^17^, endosomes^18^, and synaptic vesicles^9^. However, AO releases toxic species under intense illumination^19^. That AO property excluded the possibility of using STED imaging due to its intense laser illumination. Our studies used standard confocal illumination, thus circumventing this effect. In addition, levels of laser illumination under super-resolution microscopy would lead to loss of vesicle trapped dye obviating the possibility of dye localization within the competent vesicle.

**Fig. 4:**
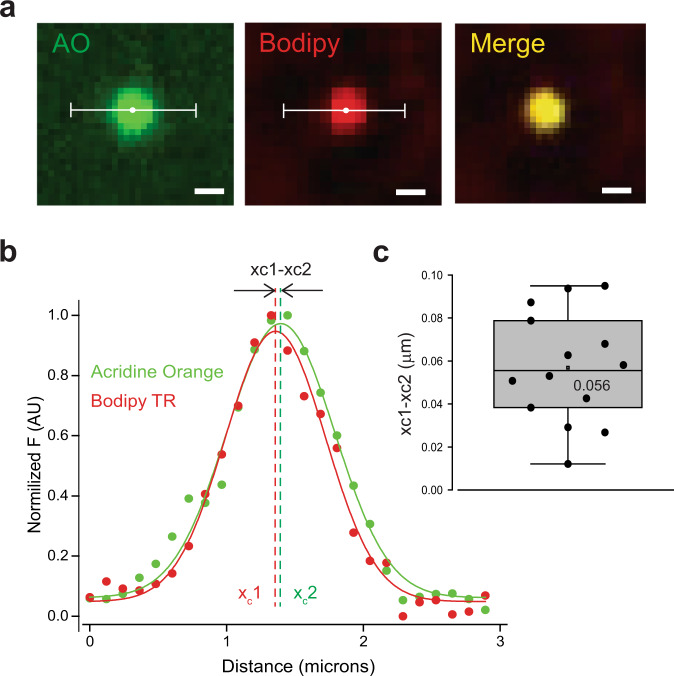
Double labeling of single EVs for content labeling and pH measurements. **a** A representative single EV confocal image on NPN membrane labeled with AO (green), Bodipy TR (red), and a merged image (yellow) (scale bar 1 µm). A bracketed line shows the analysis line cross-section for fluorescence intensity analysis. **b** A representative cross-section intensity analysis of labeled EV fluorescent spot with Gaussian fittings (solid line) of normalized data (dots) from two channels: green—AO, red—Bodipy TR. Dashed lines indicate center points (*x*_c_) of corresponding channels. **c** Summary box plot for measurements of center point difference (x_c_1−x_c_2, median 56 ± 6 nm, *n* = 14).

Recognizing the limitation that the use of laser confocal imaging significantly lowers our spatial resolution, we have applied the Gaussian fitting routine used in Fig. 3 to determine if the center of the AO fluorescent signal originates from the center of Bodipy TR labeled vesicles (Fig. 4). Fluorescent images in Fig. 4a are bracketed by a white line which delineates the vesicle cross-section yielding an intensity profile plot for both fluorescent channels (AO and Bodipy, Fig. 4b). The resultant normalized distribution was fitted using a Gaussian fitting function in order to determine the center point for each channel: x_c_1 for Bodipy TR and x_c_2 for AO detecting channels (Fig. 4b). The calculated difference between the fitted center points for each channel cross-section and the mean of the analysis was 56 ± 7 nm (Fig. 4c). Considering that the difference in center points is well below the resolution limit for our confocal imaging, we conclude that in our analyzed EVs the AO signal co-localized and originated from Bodipy TR-labeled vesicles.

### Measurement of steady-state vesicle pH

**Fig. 5:**
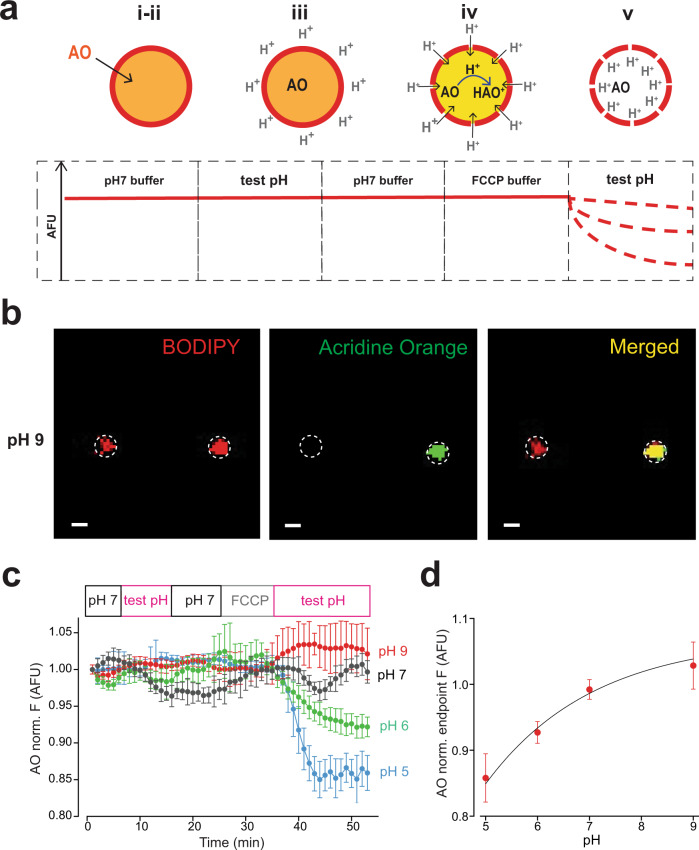
pH measurements in individual EVs. **a** Schematic of the procedure used for manipulation of internal pH inside AO-equilibrated vesicles. (i) Cell-permeant fluorescent AO in a vesicle in pH 7.4 solution. (ii) Lack of change in fluorescence of the vesicle following exposure to acidic pH solution indicates vesicle competency. (iii) Change of external solution from low pH to a neutral buffer without accompanying fluorescent change verifies membrane stabilization. (iv) Treatment with the protonophore FCCP (100 μM) in neutral buffer, followed by (v) exposure to acidic pH buffer, leading to quenching AO fluorescence signal. **b** Representative confocal microscopic image of vesicles equilibrated with 4 µM AO in buffer, pH 9, (scale bar 1 µm). Although all membranous structures were labeled with Bodipy-TR (red color, left panel), only intact vesicles loaded with AO (green fluorescence, middle panel), and only those (merged yellow color, right panel) were chosen for subsequent experimental observations. **c** Data correlating internal AO fluorescence signal with changes in vesicular pH. Freshly isolated BAL vesicles captured on NPN membrane were equilibrated in a microfluidic device with 4 µM AO solution in pH 7.4 buffer. Changes in the intra-vesicular AO fluorescence signal in response to changes in solution pH from 5 to 9 were recorded over time and analyzed with respect to the basal signal of each vesicle at pH 7.4 (normalized to 1). At pH 5, data were obtained from *n* = 233 individual (viable) vesicles from 3 independent experiments. For pH 6, a total of *n* = 263 individual, viable vesicles from 4 independent experiments were analyzed. An average fluorescence from *n* = 398 individual vesicles from 6 independent experiments was calculated for pH 7. Finally, for pH 9, *n* = 243 individual vesicles from 4 independent experiments were used. Data were adjacent averaged with a window of 5 points. **d** Dynamic range of intra-vesicular AO fluorescence signal from single vesicles. Each data point represents an average ± SEM of AO fluorescence in the last 5 min of the experiment depicted in Fig. 3c. The data were fit with a single exponential equation (*y* = *y*_0_ + *A* * ${e}^{R0* X}$).

With single vesicle size analysis in hand, we began our studies on isolated EVs by examining steady-state pH. Our initial studies were designed to assess the basal pH of isolated vesicles and utilized the pH sensitivity of AO quenching. In a series of solution changes, the leakiness or patency of vesicles was assessed as schematically depicted in Fig. 5a. Vesicles were exposed to a neutral pH buffer and then switched to an acidic buffer. Images of dye-loaded vesicles at pH 9 loaded on the chip are seen in Fig. 5b. If the AO-loaded vesicles failed to show a decrease in fluorescence when exposed to low pH solutions, they were determined to be competent and the buffer was changed back to a neutral pH. Vesicle patency was determined in the analysis of the data following the experimental acquisition. The protonophore, trifluoromethoxy carbonylcyanide phenylhydrazone (FCCP; 100 μM) was then introduced to the vesicles and followed by a sequential test pH. Data from these studies are illustrated in single vesicles (Fig. 5b) and summarized from multiple vesicles (Fig. 5c). The change in AO fluorescence as a function of pH allowed the construction of a pH calibration curve over the dynamic range of pH 5–9 (Fig. 5d). The absence of change in the AO fluorescence signal when changing external pH to 7 in the presence of the protonophore FCCP, allowed the estimation of the basal vesicle pH level to be around 7. Changes in AO fluorescence were used in all succeeding studies to determine putative NHE1 function in isolated vesicles.

### Evidence for NHE activity in EVs present in murine BAL and media from cultured HEK and J774 cells

**Fig. 6:**
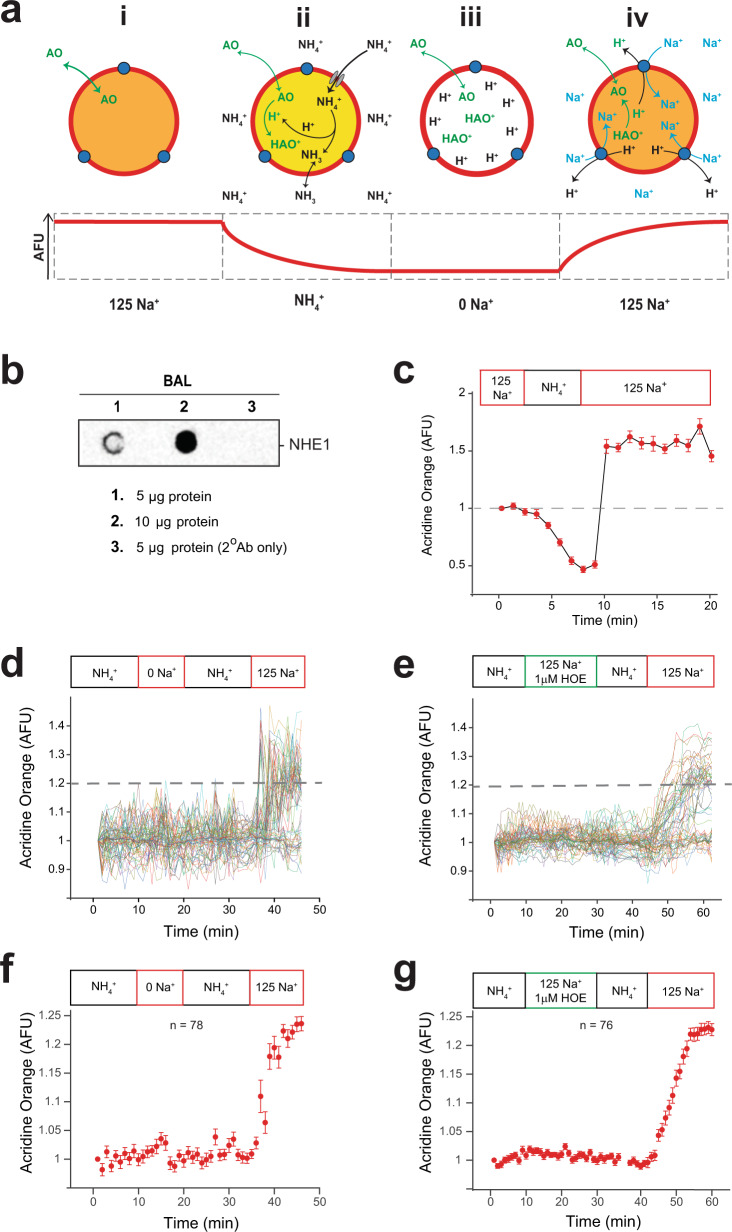
Functional characterization of EVs suggests the presence of trans-membrane NHE1 protein. **a** Schematic flow of an experiment designed to track Na+/H+ exchanger (NHE1) expression on single vesicle membranes. In a microfluidic device, (i) NPN-captured vesicles are equilibrated with AO solution in control buffer (125 mM Na+, pH 7.4), followed by (ii) acidification of vesicles with sodium-free, 50 mM ammonium chloride (NH4+) and AO solution, which produces protonation and quenching AO fluorescence, followed by (iii) wash in sodium-free, AO-containing outside buffer (pH 7.4). (iv) De-quenching of internal fluorescence by re-addition of Na+ in AO control buffer (pH 7.4) and activation of Na/H exchange. **b** A dot blot analysis demonstrates the expression of NHE1 in the sample of vesicles used for measurements. **c** Representative graph showing changes in internal AO fluorescence of vesicles, following the experimental schematic depicted above. Acidification of AO-equilibrated vesicles with 50 mM NH4 in sodium-free buffer results in a drop of internal AO fluorescence signal that is de-quenched upon re-addition of Na+ in the external buffer. **d**, **f** Alkalinization of vesicles does not occur in the absence of Na+. Change in vesicle acidification does not change in solutions that do not contain Na+ but this response is reversed in the presence of the cation in a sequential solution exchange. Not all vesicles responded to the presence of Na+ suggesting that there are likely to be three populations: vesicles that are vesicles (1) not intact, (2) intact but not functional (do not express NHE1), and (3) vesicles expressing NHE1 and are functional. The summary response in **d**, **f** was obtained from 1 chip, 1 EV isolation, *n* = 78 individual vesicles. **e**, **g** Response of vesicles to the NHE inhibitor HOE 694 (1 mM) is reversible upon sequential exposure to solutions containing Na^+^. The response to the reversible inhibitor in **e**, **g** was obtained from 1 chip, 1 EV isolation, *n* = 76 individual vesicles.

Vesicles were exposed to ammonia, a potent biological molecule NH_4_Cl (50 mM), in the presence of AO. Ammonia exists in two forms: NH_4_^+^ (ammonium ion) that dissociates into NH_3_ (ammonia) and a proton that combines with AO in the lumen of the vesicle. Both forms of ammonia appear to be membrane permeant to varying degrees depending upon cell type and membrane polarity^20, 21^ via various specific and non-specific paths^22–24^. The protonated AO is then trapped in the vesicle and quenched. Vesicles exposed sequentially to control buffer without NH_4_^+^ and absent Na^+^ remain quenched until exposure to Na^+^ containing solutions that initiate proton transport from the deprotonation of AO in exchange for Na^+^ in the extracellular solution as schematized in Fig. 6a. The presence of the exchanger NHE1 in vesicles isolated from BAL was validated in representative dot blots in Fig. 6b. The acidification of vesicles in the presence of NH_4_^+^ is illustrated in Fig. 6c as quenching of AO fluorescence that is rapidly reversed upon vesicle exposure to Na^+^ containing buffer. Vesicles that have been acidified in the presence of NH_4_^+^ and then exposed to a solution in the absence of Na^+^ or in the presence of the NHE1 inhibitor HOE-693 (Cariporide, 1 μM) in a Na^+^ containing solution failed to show dequenching in AO fluorescence (Fig. 6d, e). If, however, the acidified vesicles were then exposed to Na^+^ containing solutions, vesicles showed a clear dequenching in AO (Fig. 6d, e) characteristic of the expression of functional NHE1 protein and the reversibility of the inhibitor. Time-dependent fluorescent traces from individual vesicles seen in Fig. 6d, e reveal a distribution in responsivity with hundreds of vesicles followed simultaneously. Quantification of the individual vesicle responses is summarized in Fig. 6f, g. Only responsive vesicles were used in this analysis.

### Apparent absence of NHE activity in a subset of isolated EVs

**Fig. 7:**
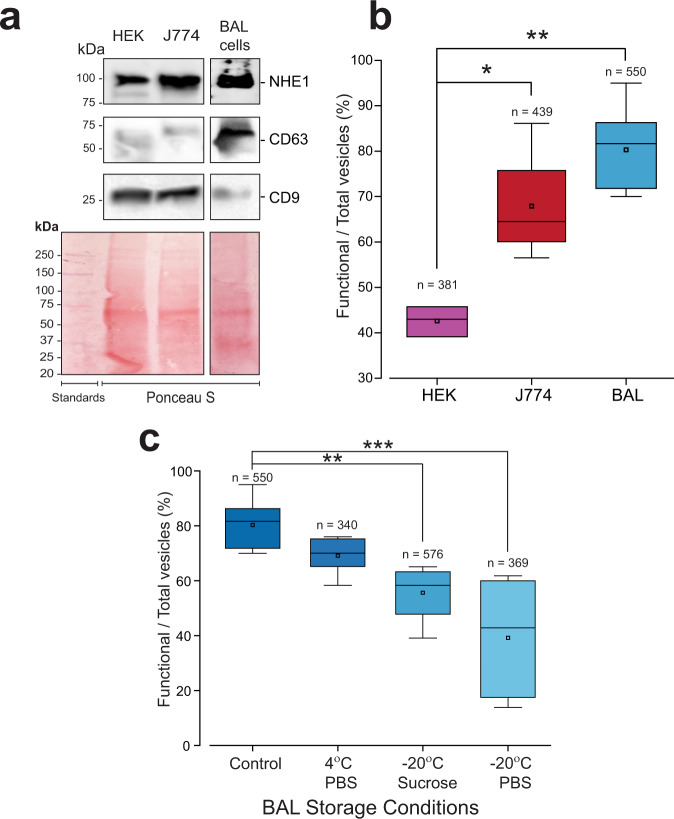
EV origin and storage affect NHE1 functionality. **a** Western blot analysis demonstrating the expression of NHE1 on multiple cell lines used experimentally: J774, HEK, and BAL cell samples. NHE1 expression is present on all cells used in the study. Specific tetraspanin expression is very dependent on cell type. CD63 is only weakly expressed on the cell lines HEK and J774. On the other hand, CD9 is weakly expressed on BAL cells but robustly expressed on the HEK and J7774 cells. **b** Comparative distribution of NHE1 functional vesicles as a percentage of the total population: HEK (3 chips, *n* = 381 individual vesicles); J774 (4 chips, *n* = 439 individual vesicles); BAL (8 chips, 12 EV isolations, *n* = 550 individual vesicles). Significance levels: *P < 0.05, **P < 0.01 (one-way ANOVA). **c** EV storage affects NHE1 functionality: comparative distribution of NHE1 functional vesicles as a percentage of the total population imaged after different storage protocols: Control (8 chips, 12 EV isolations, *n* = 550 individual vesicles); 4 °C PBS (5 chips, 10 EV isolations, *n* = 340 individual vesicles); −20 °C Sucrose (7 chips, 3 EV isolations, *n* = 576 individual vesicles); −20 °C PBS (5 chips, 2 EV isolations, *n* = 369 individual vesicles). Significance levels: **P < 0.01, ***P < 0.001 (one-way ANOVA).

A significant number of vesicles from BAL samples failed to respond to an increase in Na^+^ following NH_4_-induced acidification as evidenced in responses in Fig. 6d and e. This could be due to the absence of the NHE exchange protein in a given vesicle or to vesicle damage during isolation and storage. In order to investigate both possibilities, we compared the percentage of functionally competent vesicles with respect to NHE function in vesicles derived from culture medium from HEK cells, macrophage-like J774 cells, and finally, vesicles isolated from BAL that were derived from heterogeneity of pulmonary cell types. AF-4 analysis carried out on BAL samples suggested that the predominant vesicle radius was larger (in the range of 30–250 nm as seen in the representative analysis in Fig. S2) than that observed for vesicles derived from cultured cell media (in the range of 75-100 nm). All three EV-generating cellular sources showed expression of NHE at relatively similar levels as seen in immunoblots in Fig. 7a. Interestingly, the three cellular sources of EVs showed differential expression levels of tetraspanin CD63 and CD9, with CD63 at higher levels in BAL cells and CD9 levels higher in the cultured cells. The percentage of functional NHE expression in vesicles derived between the three cell types was also noticeably different (Fig. 7b) in acidification experiments described in Fig. 6. BAL and J774 vesicles were observed to have a higher percentage of NHE1 expressing vesicles with observable AO dequenching upon Na^+^ re-exposure than the HEK cells. Functional NHE expression was not significantly different between vesicles derived from J774 cells and BAL isolations; however, both were significantly different from HEK-derived vesicles. These differences were not noticeably reflected in immunoblot measurements of NHE1 expression in the parental cells from which the vesicles were derived (Fig. 7a) but may be due to differences in predominant size and source of vesicles (exosomes versus plasma membrane shed vesicles) not controlled for in our experiments. Vesicles derived from the plasma membrane would likely be larger, therefore, have a higher percentage of the NHE1 transport protein than exosomal vesicles derived from MVB secretion.

Finally, we used our NHE1 coupled cation transport in vesicles as a tool to examine the impact of storage conditions on BAL-derived vesicles in Fig. 7c. We compared the percentage of functional NHE1 in freshly isolated vesicles (Control) to that observed in vesicles stored in phosphate-buffered saline (PBS) at 4 °C, to vesicles stored in 100 mM sucrose at −20 °C, and to vesicles stored at −20 °C in PBS. Vesicles that underwent a freeze–thaw cycle showed a significant decrease in the percentage of NHE1 function. Although we did not explore vesicle competency, it is likely that a large fraction of vesicles undergoing freeze–thaw conditions developed leak pathways during the process of re-sealing during thawing, precluding dye loading, and acidification–alkalization responses. In addition, the freeze–thaw cycle would likely produce vesicle eversion to an inside-out membrane topology in a variable population. The inverted vesicle if competent would still carry functional NHE transport proteins that do not express a sidedness with respect to directional transport and can reverse coupled ion transport dependent entirely upon proton and Na^+^ concentration differences maintained across the vesicular membrane.

## Discussion

Exosomes have long been acknowledged as important mediators of cell-to-cell communication comprising a heterogeneous population of membrane-bound microvesicles 30–150 nm in diameter detectable in vitro as well as in vivo. Released from almost all cell types^25^ they are produced by inward budding of the endosomal compartment and are subsequently captured in MVBs targeted for plasma membrane fusion. Exosomes seen as vesicles present inside multivesicular endocytic compartments were reported in 1983 by Harding et al.^26^ and confirmed in 1985 by Pan et al.^27^. Microvesicles of roughly equivalent size (sometimes referred to as ectosomes), can be formed by budding and excision of the plasma membrane and express similar surface markers to exosomes derived from MVB membranes. Our isolation protocols co-isolate the two populations in this size range and cannot distinguish between exosomes derived from MVBs and small EVs derived from plasma membrane origin. Although isolation protocols sort vesicles allowing categorization as to size, they do not differentiate vesicles based on functionality. The investigator is blind as to the proportion of vesicles in a given isolation that have intact membranes ensuring cargo stability during biological fluid or tissue transit. Vesicle damage during preparations could impact genetic material or proteins in the lumen as well surface membrane proteins.

Our experiments provide the first assay of functional vesicles carried out at the single vesicle level. Using ultrathin (100 nm thick) NPN membranes as a capture surface, we are able to isolate vesicles from each other in the ideally sized and spaced pores of the membrane. The incorporation of the vesicle-loaded membrane chip into a microfluidic system then allowed us to quickly change the vesicle media through the backside of the porous membrane without disturbing the captured vesicles. Our approach enables simultaneous high-resolution functional imaging of hundreds of vesicles by real-time fluorescence microscopy. We use this new tool to demonstrate that many purified vesicles have an active mechanism for maintaining luminal pH and we provide a look at the heterogeneity of vesicle populations derived from different cell sources and storage based on the behavior of single EVs.

EVs may exert a paracrine effect on the surrounding tissue. This could occur either through a direct interaction between the surface proteins of EVs and the plasma membrane proteins of target cells resulting in the initiation of intracellular signaling cascades or through a transfer of the lumen-enclosed and membrane-embedded EV cargo into the cells^28^. The mechanism of fusion between EV and either the plasma membrane or endosomal membrane allowing cargo transfer remains mechanistically unclear without fusion protein interaction. Due to their low immunogenicity, stability in the bodily fluids, and the presence of specific surface receptors, EVs can home to specific targets in the body, making them a potentially attractive carrier of therapeutic payload^29^. Endogenous EVs isolated from some tissues or cells, such as mesenchymal stem cells, are known to have immunosuppressive and regenerative properties on their own and have been successfully used in acute respiratory distress syndrome therapy^30^. However, more general use of EVs, as vehicles for the delivery of therapeutics, requires the engineering of EVs to incorporate specific proteins and miRNAs. A platform designed to study single vesicle function with respect to luminal content as well as immunogenic surface protein spectrum is important to the engineering design and proposed feasibility of EVs as effective and efficient translation agents in disease treatment.

As mediators of cell–cell function, EVs must protect the specific mRNAs, regulatory microRNAs, lipids, and proteins over uncharted distances in changing biological fluids, facilitating intercellular communication. EVs have, in general, properties shared by coated viruses that optimize their properties for transferring genetic material into tissues. Recognized architectural advantages of the nanocarriers include a relatively small size for tissue penetration^31^, a negative zeta potential that facilitates an extended period of circulation versus aggregation^32, 33^, and a flexible surface structure also enhancing unimpeded transit in narrow spaces^34^. In addition, EVs appear to evade clearance by the immune system that leading to degradation^35^. The design of our single vesicle visualization platform will allow the study of vesicles and their interactions with viral elements which are approximately the same size. These studies may provide the possibility for insight into how EVs might act as an innate antiviral mechanism.

In their role as transporters of genetic cargo, EVs would be obliged to maintain a neutral pH similar to that in the cytoplasmic milieu from which the cargo is derived. We surmised that this stabilization of the luminal compartment with respect to pH would require and dictate processes similar to that which maintains cytoplasmic pH at neutrality in cells and or tissues that face swings in extracellular pH during organellar function. Examples include renal tubule cells and the unusual acid-base environment of the colon. The ubiquitously expressed Na^+^/H^+^ exchanger, NHE1, localized to the plasma membrane, catalyzes an electroneutral exchange of extracellular Na^+^ for intracellular H^+^ thereby controlling both intracellular pH and cell volume in cells throughout the phylogenetic tree^36^. The importance of NHE1 in pH homeostasis underlies a diversity of cellular functions including cell migration and growth rate as well as division and differentiation which are dependent upon the proton fluxes driven in part by members of the NHE family^36^. The fact that NHE1 activation produces a rapid increase in cellular pH and protects against intracellular acidification in cells as diverse as neurons^37^ and bone-forming osteoblasts^38^ provided us with a strong candidate for EV pH regulation.

Our examination of mechanisms maintaining acid-base balance in EVs was inherently limited by the fact that vesicles are not homogeneous and cannot be isolated in large enough populations to facilitate examination using standard biochemical techniques. This drawback drove the development of a stabilizing platform to study individual vesicles in a microfluidics environment enabling the possibility to study the steady-state pH of vesicles in the presence of changing environmental pH as well as determine whether the ubiquitously expressed NHE1 was active in the EV pH regulatory process. The microfluidic device enabled the imaging of the molecular function of plasma membrane proteins at the single-EV level. Our data clearly show that at a steady state the luminal pH is at neutrality and, furthermore, NHE1 is present at the EV surface and active in the role of maintaining a neutral luminal environment. NHE1 then plays a crucial role in stabilizing genetic cargo transport in the face of a changing biological fluid composition.

## Methods

### Animals

The Animal Care and Use Committee at the University of Chicago approved all of the procedures outlined here. All animals were housed in a specific, pathogen-free, biohazard level 2 facility, maintained by The University of Chicago Animal Resources Center (Chicago, IL). Animal genotyping was performed by Transnetyx, Inc., (Cordova, TN).

### NPN membranes

Chips with NPN membranes were purchased from SiMPore Inc. (West Henrietta, NY). Each chip was 300 µm thick, 5.4 mm wide and 5.4 mm long, and contained an active, 100 nm-thick NPN membrane with dimensions of 0.7 mm width and 2 mm length. Each nanomembrane was manufactured with ~5 × 10^7^ pores with dimensions ranging from 30 to 140 nm using manufacturing conditions that allow for some pore merger events.^39^ Vesicles in this size range become trapped inside of the tapered pores or on top of pores, while smaller vesicles and protein aggregates will pass through.

### Device fabrication

Microfluidic devices were fabricated using PDMS sheets (Trelleborg Sealing Solutions Americas, Fort Wayne, IN). Custom NuSil 4930 300 µm-thick silicone sheets were patterned using a conventional digital desktop cutter (Silhouette America, Oren, UT). The top 3 layers of the device, including the 100 µm wide channel, the sealing layer, and the PDMS slab were bonded to each other using UV/ozone treatment. The middle compartment was cut out from this assembly (6 mm × 6 mm); this middle compartment would allow for NPN chip placement after it was loaded with vesicles. The top assembly was then stacked on top of the bottom NPN housing layer using the alignment marks. Then, inlets and outlets were punched out. Finally, the PDMS assembly was bonded to a glass slide using UV/ozone treatment. For final device assembly, the NPN chip was then loaded in the EV capture section and placed within the NPN housing layer via the middle cutout compartment. The compartment was then sealed shut using PDMS as adhesive and the inlets and outlets were filled with aqueous media. The finished device was placed in an oven for 20 min at 40 °C to cure the PDMS.

### Procurement of EV samples

EV samples were obtained from two sources, BAL fluids and conditioned media from cells lines in culture. BAL fluids were collected from mice using standard methods, as previously described^40^. After harvesting, the BAL fluids were centrifuged at 300 × *g* for 5 min to separate cells from the fluid. The cell pellet was frozen at 20 °C for downstream protein analysis. We then used a series of high-speed centrifugation of supernatant to concentrate EVs while also removing large debris and dead cells (2000 × *g* for 10 min to remove dead cells; 10,000 × *g* for 40 min to remove large debris; 4000 × *g* for 10 min in Amicon (Millipore Sigma) spin columns to concentrate vesicle samples). The final sample was between 200 and 500 µl and used for EV capture on plasma cleaned NPN membranes.

Media from cultured cell lines were obtained from HEK and J774 cells obtained from the American Type Culture Collection (ATCC) and cultured in EV-depleted DMEM supplemented with 10% FBS, 1% (vol/vol) penicillin–streptomycin. All cultures were maintained at 37 °C, 5% CO_2_, and 100% humidity. Cells were grown to 80% confluence in T75 flasks. At this point, the medium was removed and the cells were passaged either for future studies or trypsinized for cell protein analysis. The media was subjected to the same high-speed centrifugation cycle as the BAL fluids before concentrating to a final volume between 200 and 500 µl.

The EVs shown in the EM image in Fig. 1b were purchased from Hansa Biomed. They were plasma-derived EVs at 100 μg protein content (expected) at a concentration of approximately 10 × 10^10^/ml. They were reconstituted in water to reduce the total amount of salts then spun against the NPN membrane for facilitated capture at 500 × *g* for 30 min. The sample was then extracted from the SepCon unit, dehydrated in a sequential ethanol dilution series (50%-100% EtOH) for 10 min at each stage. The sample was then coated with ~10 nm gold before imaging in a Zeiss Auriga SEM at 10 kV.

### EV loading onto NPN membranes

Loading of the sample fluid onto the NPN membrane was achieved by the use of SepCon™ centrifuge units (SiMPore Inc., West Henrietta, NY). The centrifuge units contained a housing area where the NPN membranes were placed. The NPN membrane was pre-treated to facilitate wetting by either exposing to UV/ozone treatment 20–30 min before loading or wetting the backside through a port provided in the SepCon unit. The range of plasma conditions tried for NPN preparation did not make an obvious difference in the wettability of the membranes or the number of vesicles captured, although this was not rigorously quantified. Once in place and pre-treated, 120 µl of the sample was deposited on top of the membrane. The sample was then spun at 3000 × *g* for 5 min trapping EVs within the nanopores. The NPN membrane-containing chip was then removed from the centrifuge unit and placed on the microfluidic platform. The device assembled on the stage of laser scanning confocal microscope allowed for sequential exposure of the captured EVs to buffer solutions. Inlet and outlet tubing allowed for the flow of solutions over the NPN membrane-immobilized vesicles, at a rate of 5–10 µl/min, maintained by a pressure-driven microfluidic flow system (ElveFlow®) coupled with a flow rate control system. Our measurements were taken under steady-state flow conditions. In similar devices for other studies used under constant flow rate, the pressure settles to a steady-state level within a minute of the onset of flow or solutions^41^. Thus, we presume that our experiments reach steady flow in under a minute which is much faster than the ~10 min measurement windows used to acquire data. Different treatment windows were tried and were not found to make any obvious difference, although we did not quantify the difference. Once the microfluidic device was placed on the microscope for imaging, NPN membrane-immobilized vesicles were equilibrated for 5–10 min before they were exposed to any stimuli, and images were taken. It is important to note, our experiments were conducted under constant pressure and, therefore, stable flow conditions. Solution changes were made such that all transient fluctuations were 10 min before the peak amplitude in response was determined and averaged over 5 time points.

### EV isolation using size-exclusion column chromatography

To obtain a high purity sample of EVs, we used a size-exclusion chromatography column (Izon Science, Christchurch, New Zealand) to isolate the EV fractions of interest while separating out free-floating proteins. Eluate fractions (~500 μl per fraction) were collected individually; fraction numbers 6–10 contained most of the EV content and were pooled and concentrated to a final volume between 200 and 300 µl using Amicon 100 kDa molecular weight cut-off (MWCO) centrifugation filters (Millipore Sigma).

### EV isolation using AF4

A higher resolution of vesicle size distribution as compared to that obtained with the size exclusion column strategy was obtained with the technique of AF4 (Wyatt Eclipse with Dilution Control Module, Wyatt Technology, Santa Barbara, CA). With AF4 separation we are able to concentrate, quantitate, characterize, and elute EVs, separate from contaminants, in real time with virtually no retention of contaminating soluble protein. A multitude of detectors including multi-angle light scattering, DLS, and ultraviolet gave reproducible MW measurements.

### EV characterization and labeling

Size-exclusion chromatography prepared EVs were analyzed for membrane markers and size. EV content of isolated fractions was confirmed by protein analysis (Qubit, Thermo Fisher Scientific), and EV size and quantity was performed by NanoSight Nanoparticle Tracking Analysis (Malvern | PANalytical Products, Westborough, MA).

Immunoblotting analysis validated the presence of known EV membrane markers CD63 and CD9. Additionally, the lipid fluorescent label Bodipy TR (1 µM) was used to label the outer lipid structure of EVs for visualization with confocal microscopy.

### Laser confocal live EV time-lapse imaging

The microfluidic platforms were sealed and loaded with the NPN chip, the inlets and outlets of the device were connected to a pressure-driven microfluidic flow control system (ElveFlow, Paris, FR). The flow rate was kept between 5 and 10 µl/min for the duration of the experiment. The microfluidic device was placed on the microscope for imaging. Control fluid flow was administered for equilibration for 5–10 min while EVs were loaded with the fluorescent dye AO before the initiation of image acquisition. Changes in intravesicular pH were monitored using AO (4 µM). EVs were pretreated with a 50 mM NH_4_^+^ solution to artificially acidify the lumen of membrane competent, sealed vesicles visualized on the chip with the fluorescent lipid dye, Bodipy TR. The fluid medium was switched after a 15 min control period to a desired ionic condition always keeping the AO concentration constant. The solution ionic conditions varied from a high Na^+^ solution, a Na^+^-free solution, or a high Na^+^ solution containing the NHE1 inhibitor, HOE (cariporide, 1 µM). In the pH experiments, solutions differing in pH were introduced into the device at a volumetric flow rate of 10 µl/min. Image acquisition, processing, and analysis were performed at the University of Chicago Integrated Light Microscopy Facility. Images were captured with a Leica SP5 Tandem Scanner Spectral 2-photon confocal microscope. Image processing was performed using Bitplane Imaris software v. 9.1.2 (Andor Technology PLC, Belfast, N. Ireland).

### Super-resolution STED EV imaging and analysis

Super-resolution STED imaging was done using a Leica SP8 laser confocal microscope with STED functionality. STED depletion was performed with 775 nm laser set at 100% power. STED images were analyzed using the Leica LIGHTNING (Lng) image deconvolution technique and further analyzed using the ImageJ analysis software.

### EV DLS size analysis

DLS EV size measurements were performed on AF4 separated vesicular fractions using the DynaPro DLS plate reader (Wyatt Technology Corp., Santa Barbara, CA) in 96-well plates. The DLS data were analyzed using Dynamics version 7.8.1.3. software (Wyatt Technology Corp).

### Solutions

Solutions used in the functional analysis of vesicles were as follows:

High Na^+^ solution contained (in mM): 125 NaCl, 2.5 KCl, 10 HEPES, 1.5 MgCl_2_, 2.5 CaCl_2_, 10 glucose, 20 sucrose, pH 7.4. Na^+^-free solution contained (in mM): 125 *N*-methyl d-glucamine, 40 HCl, 10 HEPES, 1.5 MgCl_2_, 2.5 CaCl_2_, 10 glucose, 20 sucrose; pH 7.4. Acid-loading solution contained (in mM): 50 NH_4_Cl, 30 KCl, 10 K methane sulfonate (MeSO_4_), 10 HEPES, pH 7.4.

For pH measurements, we used an Intracellular pH calibration buffer Kit (Invitrogen, P35379). In all cases, the osmolarity was set to 300 ± 10 mOsm with the major salt.

### Dot blot development and analysis

BAL from 3 mice was pooled together (~9 ml) and cleared by one low and two medium-speed centrifugations to remove live cells, dead cells, and cellular debris, as described above, followed by concentration down to ~500 µl in Amicon Ultra-15 Centrifugal Filters (Millipore Sigma) with a MWCO of 100 kDa.

Concentrated BAL was lysed on ice by passaging 10 times through a 30G1 needle (BD) in 10× RIPA buffer (Sigma), supplemented with cOmplete™ Protease Inhibitor Cocktail (Millipore Sigma). The lysate was further cleared from insolubilized components by centrifugation at 17,000 × *g* for 10 min in a tabletop centrifuge (Fisher Scientific).

BAL samples were blotted onto methanol-activated PVDF membrane (BioRad), using a Minifold-I Dot-Blot System (Schleicher and Schuell BioScience Inc., Keene N.H.). Briefly, 200 µl samples were added to each well of the manifold wells and allowed to passively filter through by gravity flow for ~2 h, followed by blocking using gravity flow with 1% bovine serum albumin (BSA; Sigma-Aldrich) solution in TBS buffer (20 mM Tris and 150 mM NaCl) for 1 h. Blocked samples were washed with 500 µl of TBS-T solution (0.1% Tween-20 in TBS) 3 times by vacuum suction, after which the PVDF membrane was removed from the manifold and washed additional three times with TBS-T for 5 min in a plastic box on a rocker (Cole Parmer). Primary antibodies, diluted 1:3000 in 0.1% BSA (Sigma-Aldrich) in TBS-T, were incubated with the membrane for 1 h at room temperature (RT), followed by three washes for 10 min with TBS-T solution. Horseradish peroxidase (HRP)-conjugated secondary antibodies, at 1:150,000 dilution, were incubated with the membrane for an hour at RT, followed by three washes with TBS-T for 10 min and an additional wash in TBS-T for 30 min. Dot Blots were developed using Super Signal West Pico PLUS HRP Substrate (Thermo Scientific) and detected by Chemidoc (Azure Biosystems, Dublin, CA.).

### Western blot

Mammalian cells cultured near confluence in T75 flasks were washed twice with cold PBS and scraped into 200 µl of Pierce 1x RIPA buffer (Thermo Scientific), supplemented with protease inhibitor cocktail (Millipore Sigma), and then transferred to 1.5 ml microtube kept on ice. Cells were vortexed briefly and disrupted by trituration 10× with a 23 G ¼ inch syringe needle (Becton, Dickinson and Co. Thermo Scientific). To remove unbroken cells and cellular debris, lysates were spun at 17,000 × *g* for 10 min and the supernatant was collected for downstream applications, as well as for determination of protein concentration by the BCA Protein Assay Kit (Thermo Scientific).

For running Western blots, 30 µg of cell lysates in Sample Buffer was loaded per each well of 4–20% Precast Protein Gel (BioRad, Hercules, CA) and then transferred to PVDF membrane. For blocking, 3% BSA (Sigma-Aldrich) in TBS buffer was used, while antibodies were diluted in 1% BSA in TBS-T. For detection of CD63 protein, we used either anti-mouse CD63 (ab217345) or anti-human CD63 (ab134045) (Abcam, Waltham, MA); for detection of NHE1, the Anti-Na+/H+ Exchanger 1 (extracellular) was used from Alomone Labs (ANX-010, Jerusalem, Isreal); for secondary antibodies, the HRP-conjugated goat anti-rabbit IgG H&L (ab6721, Abcam) was used; primary antibodies were used at 1:2000 and secondary antibodies at 1:10,000 dilutions.

### Statistics and reproducibility

To analyze the statistically significant difference between the groups, ANOVA analyses (Student’s *t* test and Mann–Whitney Rank-Sum Test) were used. Data are represented as mean ± SEM. In all studies, a chip represented an independent experiment. At least three mice were used in all experimental studies.

### Reporting summary

Further information on research design is available in the Nature Research Reporting Summary linked to this article.

## Supplementary information

Supplementary Information

Description of Additional Supplementary Files

Supplementary Data 1

Reporting Summary

## Data Availability

The authors declare that the data supporting the findings of this study are available within the paper and its Supplementary Information files. All source data underlying the graphs shown in the main and Supplementary Figures are presented in Supplementary Data 1.
