# Supplementary material for: Real time imaging of single extracellular vesicle pH regulation in a microfluidic cross-flow filtration platform
Source: Commun Biol. 2022 Jan 10;5:13. doi: 10.1038/s42003-021-02965-7 (PMC8748679; doi:10.1038/s42003-021-02965-7)
Supplement: Supplementary file 1 — Supplementary Information [file 42003_2021_2965_MOESM1_ESM.pdf]

Supplementary information for

Real time imaging of single extracellular vesicle pH regulation in a microfluidic cross-flow filtration platform

Vladimir Riazanski[1], Gerardo Mauleon[1], Kilean Lucas[2], Samuel Walker[2],

Adriana M. Zimnicka[1], James L. McGrath[2*], Deborah J. Nelson[1*]

[1]Department of Pharmacological and Physiological Sciences
The University of Chicago
Chicago, IL 60637

[2]Department of Biomedical Engineering
University of Rochester
Rochester, N.Y. 14627

[*]These authors jointly supervised this work

Corresponding author email: nelson@uchicago.edu

Supplementary Figures

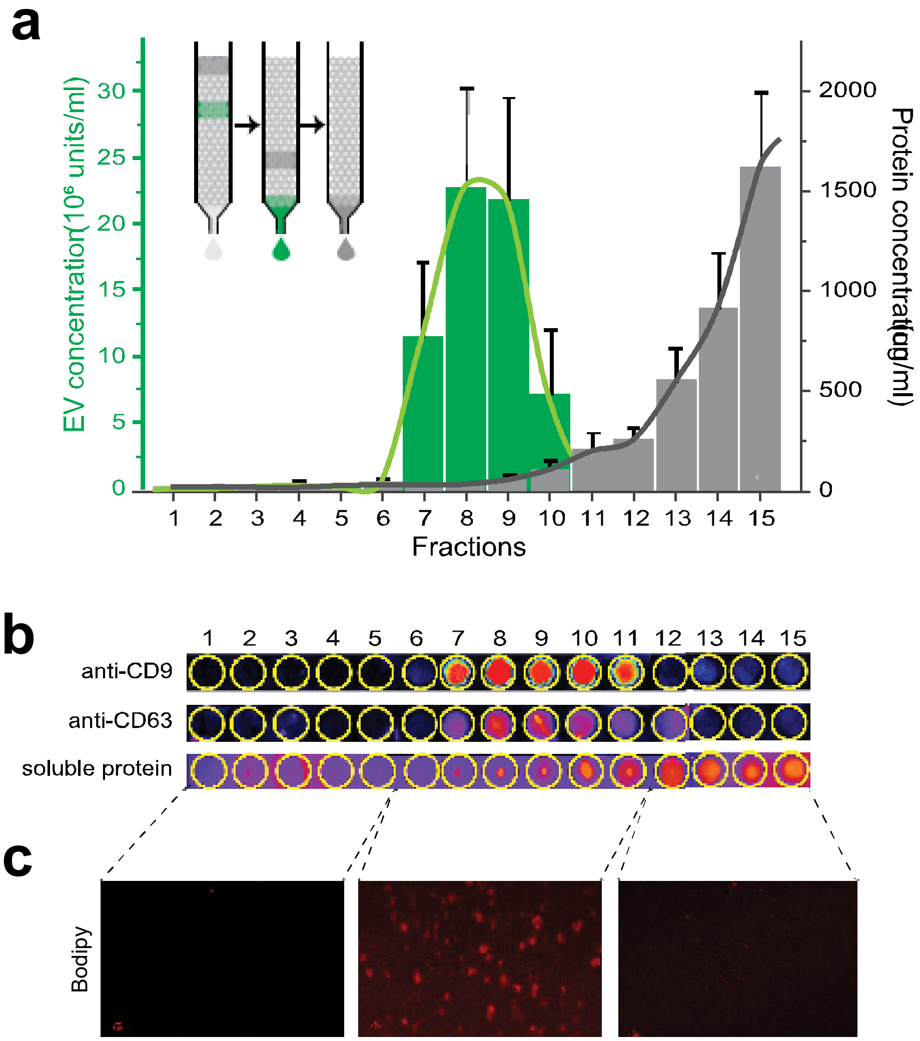

Figure S1. Biocharacteristics of EVs.

a. Size-exclusion chromatography following high-speed centrifugation steps separate EVs from free-floating protein in biological fluids (top left schematic). The efficacy of EV isolation from cell media can be demonstrated through NanoSight measurements indicating the number of particles measured in each fraction (green bars) compared to Qubit protein analysis (gray bars). The vesicles in fractions 7 to 10 have a median size of 109.5 nm as determined by NanoSight analysis. **b**. A dot blot analysis illustrates the fractions where tetraspanins CD9 and CD63 are situated compared to free-floating protein (reversible protein stain (Pierce, Thermo Scientific)). **c**. Confocal microscopy images showing Bodipy TR labelling of EVs in expected fractions.

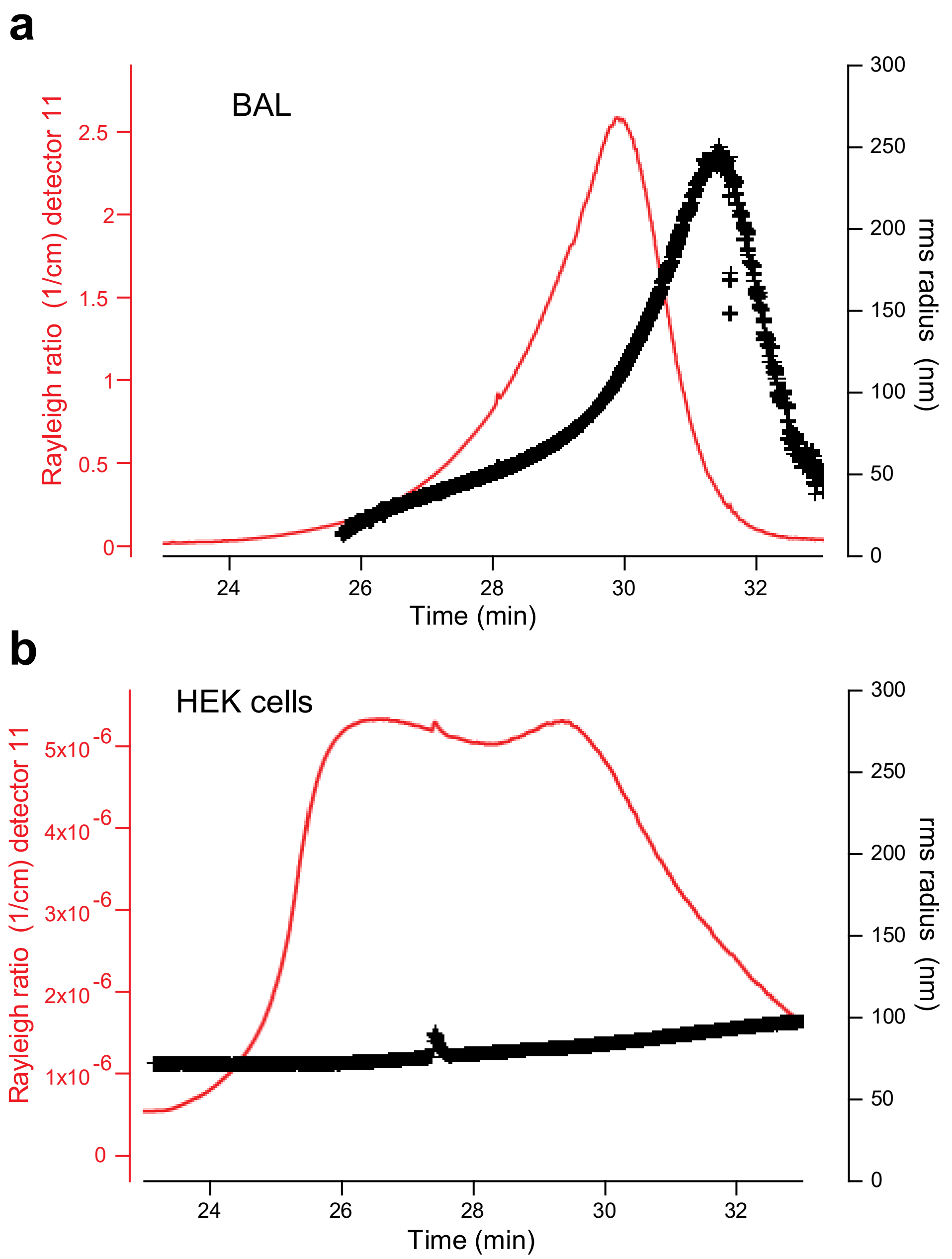

Figure S2. Different EV sizes from two sources determined by AF4 separation.

a. A representative an AF4 run fractogram profile of a mouse BAL preparation. Solid red line shows a time course of dynamic light scattering Reyleigth ratio changes during an injection of 100 µl of BAL sample. Black trace represents hydrodynamic radius (rms radius) changes during the time course of the sample injection. **b.** A representative an AF4 run fractogram profile of a 100 µl sample from HEK293T cell culture grown in a CELLine 1000 Bioreactor (Integra BioSciences).

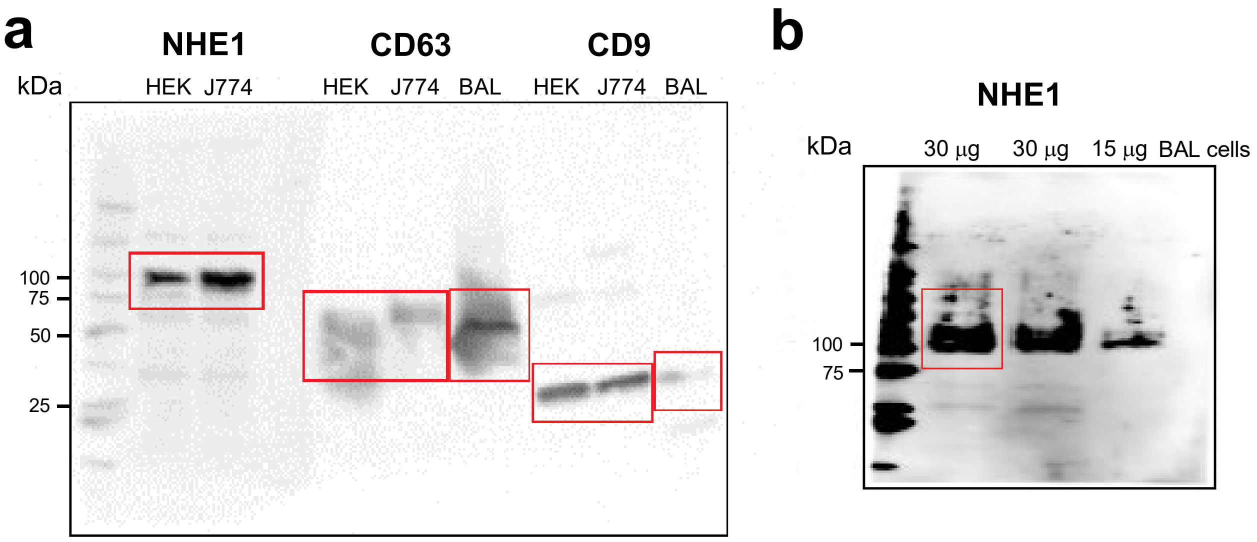

Figure S3. Full length Western blot images used for cropped image in Fig.7a .

Full images of Western blots demonstrating the presence of: **a** - NHE1 protein, CD9 and CD63 tetraspanin protein expression in cell lines: HEK293T (HEK), J774A.1 (J774) and BAL cell lysate (BAL). Cell lysate containing 30 µg of protein in Sample Buffer was loaded in each well. **b** – NHE1 protein in BAL cell lysate loaded with different protein concentrations per well. Red rectangle – cropped portion of the image used in Fig.7a.
