# Supplementary material for: Real time imaging of single extracellular vesicle pH regulation in a microfluidic cross-flow filtration platform
Source: Commun Biol. 2022 Jan 10;5:13. doi: 10.1038/s42003-021-02965-7 (PMC8748679; doi:10.1038/s42003-021-02965-7)
Supplement: Supplementary file 2 — Description of Additional Supplementary Files [file 42003_2021_2965_MOESM2_ESM.pdf]

Description of Additional Supplementary Files

File name: Supplementary Data 1

Description: All source data underlying the graphs in the main and supplementary figures.
